# Nrf1 and Nrf2 Transcription Factors Regulate Androgen Receptor Transactivation in Prostate Cancer Cells

**DOI:** 10.1371/journal.pone.0087204

**Published:** 2014-01-22

**Authors:** Michelle A. Schultz, Sharika S. Hagan, Amrita Datta, Yiguo Zhang, Michael L. Freeman, Suresh C. Sikka, Asim B. Abdel-Mageed, Debasis Mondal

**Affiliations:** 1 Department of Pharmacology, Tulane University School of Medicine, New Orleans, Louisiana, United States of America; 2 Department of Urology, Tulane University School of Medicine, New Orleans, Louisiana, United States of America; 3 Laboratory of Cell Biochemistry and Gene Regulation, University of Chongqing, Chongqing, China; 4 Department of Radiation Oncology, Vanderbilt University Medical Center, Nashville, Tennessee, United States of America; Institut de Génomique Fonctionnelle de Lyon, France

## Abstract

Despite androgen deprivation therapy (ADT), persistent androgen receptor (AR) signaling enables outgrowth of castration resistant prostate cancer (CRPC). In prostate cancer (PCa) cells, ADT may enhance AR activity through induction of oxidative stress. Herein, we investigated the roles of Nrf1 and Nrf2, transcription factors that regulate antioxidant gene expression, on hormone-mediated AR transactivation using a syngeneic *in vitro* model of androgen dependent (LNCaP) and castration resistant (C4-2B) PCa cells. Dihydrotestosterone (DHT) stimulated transactivation of the androgen response element (ARE) was significantly greater in C4-2B cells than in LNCaP cells. DHT-induced AR transactivation was coupled with higher nuclear translocation of p65-Nrf1 in C4-2B cells, as compared to LNCaP cells. Conversely, DHT stimulation suppressed total Nrf2 levels in C4-2B cells but elevated total Nrf2 levels in LNCaP cells. Interestingly, siRNA mediated silencing of Nrf1 attenuated AR transactivation while p65-Nrf1 overexpression enhanced AR transactivation. Subsequent studies showed that Nrf1 physically interacts with AR and enhances AR’s DNA-binding activity, suggesting that the p65-Nrf1 isoform is a potential AR coactivator. In contrast, Nrf2 suppressed AR-mediated transactivation by stimulating the nuclear accumulation of the p120-Nrf1 which suppressed AR transactivation. Quantitative RT-PCR studies further validated the inductive effects of p65-Nrf1 isoform on the androgen regulated genes, PSA and TMPRSS2. Therefore, our findings implicate differential roles of Nrf1 and Nrf2 in regulating AR transactivation in PCa cells. Our findings also indicate that the DHT-stimulated increase in p65-Nrf1 and the simultaneous suppression of both Nrf2 and p120-Nrf1 ultimately facilitates AR transactivation in CRPC cells.

## Introduction

Prostate cancer (PCa) is the second leading cause of cancer related deaths in American men [1] and elevated androgen receptor (AR) signaling facilitates PCa growth. Hence, androgen deprivation therapy (ADT) was designed to deplete systemic androgen levels and thus suppress AR signaling in hormone dependent PCa cells [2]. However, patients only respond to ADT for approximately 18 months due to the selection and outgrowth of castration resistant prostate cancer (CRPC) cells. Interestingly, CRPC cells retain both AR expression and function [2], [3]. Therefore, understanding the mechanisms of persistent AR function in CRPC cells despite ADT will aid in developing therapeutic strategies that suppress PCa recurrence.

It has been suggested that residual androgen production within the tumor microenvironment contributes to persistent AR signaling [3]. Dihydrotestosterone (DHT) is a potent androgen that stimulates AR mediated transactivation at the androgen response element (ARE), present on promoters of numerous genes important in PCa cell growth [4]. Interestingly, the classical AR transactivation pathway is often bypassed in CRPC cells where persistent AR function occurs despite low androgen levels [5], [6]. This AR transactivation in CRPC cells has been attributed to increased AR expression and enhanced expression of enzymes that convert androgens to DHT [3], [7]. However, recent evidences also suggest that parallel signaling pathways that increase the expression and activity of AR coactivators may play a significant role in regulating AR activity [3], [8]. Some of these AR coactivators may change the conformation of AR ligand binding pocket, thus increasing the binding specificity of AR to steroid ligands. Alternatively, AR may associate with various cofactors and chaperones that facilitate its nuclear localization and ARE binding capacity [9]. Therefore, the identification of AR cofactors will improve our understanding of PCa progression to CRPC.

Studies have shown that ADT can induce oxidative stress and reactive oxygen species (ROS) play a significant role in PCa progression to castration resistance [10]. Chronic oxidative stress has been observed in aggressive PCa cells and reports have demonstrated that these cells can utilize ROS induced antioxidant proteins to enhance survival and maintain AR signaling [6], [11]–[13]. Indeed, many effectors of ROS signaling that function as AR coactivators are overexpressed in PCa and their expression can be regulated by hormone signaling [14]–[16]. The antioxidant protein peroxiredoxin-1 (Prx-1) acts as a chaperone to enhance hormone signaling and androgen sensitivity via direct interaction with AR, which augments its nuclear localization [14], [15]. Furthermore, disruption of androgen signaling (i.e. ADT) in the prostate can induce oxidative stress by increasing the expression of ROS producing NADPH oxidases (NOX) [16], [17]. These changes in ROS affect the activity of transcription factors such as Nrf1 and Nrf2 (NF-E2 related factor 1 and 2) that that regulate the expression of numerous antioxidant proteins and NADPH Oxidases [18]–[20]. The resultant changes in NOX and antioxidant protein expression may be associated with increased tumor survival [21]–[23]. However, although both Nrf1 and Nrf2 have significant effects on oxidative stress signaling, their direct effects on AR transactivation have not been previously investigated.

Nrf1 and Nrf2 are master regulators of oxidative stress induced gene expression [18], [24]–[26]. They are cap-n-collar basic leucine zipper (CNC-bZIP) transcription factors that, in response to various forms of oxidative stress, can regulate gene expression through the electrophile response element (EpRE). Under normal homeostatic redox conditions, Nrf2 is sequestered by Keap1 (Kelch-like ECH-associated protein 1) in the cytoplasm where it negatively regulates Nrf2 through ubiquitin mediated proteasomal degradation [26]. Upon ROS stimulation, Keap1 releases Nrf2 to permit its nuclear localization and transactivation via the EpRE sequences. However, although much attention has been focused on the role of Nrf2 in cancer [25], investigations on the role of Nrf1 has been severely lacking.

In contrast to Nrf2, the N-terminal domain (NTD) of Nrf1 (TCF11), which anchors Nrf1 to the endoplasmic reticulum (ER) membrane and the nuclear membrane, regulates Nrf1 activation and its translocation to the nucleus [27]–[29]. Furthermore, the human Nrf1 gene can generate both full length 120 kDa Nrf1 (p120-Nrf1) and several truncated (36, 55, 65, and 95 kDa) isoforms of Nrf1 [30], [31]. Of these smaller Nrf1 isoforms, the N-terminal-truncated 65 kDa isoform (p65-Nrf1) has been shown to possess significant regulatory effects with regard to Nrf2 mediated transcription. Interestingly, enforced expression of p65-Nrf1 can inhibit Nrf2 mediated induction of EpRE-regulated genes [30]. Studies have also indicated that both Nrf1 and Nrf2 mediate ROS signaling [18], [30]. Thus, ROS signaling and cellular homeostasis can occur via the regulation of a critical balance between Nrf1 and Nrf2 expression and activity. However, their role in AR transactivation in PCa cells has not been investigated.

Several studies have implicated the importance of Nrf2 in PCa [32]–[34]. The expression of Nrf2 is negatively correlated with Gleason scores in PCa patients [32] and reduced levels of Nrf2 have been linked to increased aggressiveness in the TRAMP PCa mouse model [33], [34]. Interestingly, it has also been suggested that Nrf1 can regulate Nrf2 expression through regulation of an EpRE located in the Nrf2 promoter region [35]. However, despite the ability of Nrf1 to repress Nrf2 mediated transcription [30], the role of Nrf1 in PCa progression is unknown. Our previous investigations have shown that the CRPC cell line C4-2B has elevated expression of p65-Nrf1 and decreased expression of Nrf2, as compared to the androgen dependent LNCaP cells and the non-tumorigenic RWPE-1 and RWPE-2 cells [36]. Since both enhanced AR signaling and androgen deprivation can induce ROS expression, we postulated that Nrf1 and Nrf2 may play a direct role in regulating AR signaling in PCa cells [3], [10]. Therefore, we investigated whether Nrf1 and Nrf2 differentially modulate AR transactivation in androgen dependent LNCaP cells and in their androgen independent sub-line, C4-2B.

Since LNCaP and C4-2B cells are syngeneic PCa lines, our findings in these cell lines indicate that Nrf1 and Nrf2 may have significant roles in PCa progression through the manifestation of CRPC phenotype. Our findings clearly showed that the opposing functions of Nrf2 and the p65 and p120 isoforms of Nrf1 can regulate DHT-induced AR transactivation in PCa cells. Our studies further implicate novel mechanisms via which Nrf1 and Nrf2 regulation is modified in the castration resistant C4-2B cell line to facilitate the enhanced AR transactivation.

## Materials and Methods

### Cell Culture

LNCaP cells were obtained from American Type Culture Collection (ATCC; Catalog # CRL-1740). The C4-2B cell line is a bone metastatic subline derived from LNCaP, and was a kind gift from Dr. Lelund Chung [37]. Both cell lines were cultured in RPMI-1640 media from Mediatech (Manassas, VA) supplemented with 10% fetal bovine serum (FBS) from Atlanta Biologicals (Lawrenceville, GA) and 1% penicillin/streptomycin (Mediatech). DHT treatments were carried out in phenol red free RPMI media (Mediatech) with 10% charcoal stripped fetal bovine serum (CS-FBS) from Innovative Research labs (Novi, Michigan). Cells were trypsinized, plated and allowed to attach overnight in complete media, following which media was changed to CS-FBS containing RPMI. Following overnight incubation in CS-FBS containing media, cells were exposed to DHT (0–10 nM) in CS-FBS media and harvested at the indicated times to measure RNA, protein, and reporter gene expression.

### Reagents

DHT was obtained from Steraloids (Wilton, VA). Nrf1 antibody was obtained from Proteintech Group (Chicago, IL). Antibodies to Nrf2, AR, and TATA Binding Protein (TBP) were obtained from Abcam (Cambridge, MA). Isotype IgG (non-specific control) and all secondary anti-human antibodies were obtained from Santa Cruz (Santa Cruz, CA). Anti-V5 antibody was obtained from Invitrogen (Carlsbad, CA). The AR antibody used for ChIP assays was obtained from Active Motif (Carlsbad, CA). The Nrf1 specific siRNA and the non-specific control (NC1) siRNA were obtained from Integrated DNA Technologies (Coralville, IA; Cat# HSC.RNAI. N003204.12.1-3). Plasmids were obtained from the following sources: p65-Nrf1 expression vector (p65-Nrf1-V5His) was a gracious gift from Dr. Chan [30], the p120-Nrf1-V5His expression vector was obtained from Dr. Zhang [38] and the psPSA-Luc reporter (firefly luciferase) vector was obtained from Dr. Abdel-Mageed’s laboratory [39]. The pRL-TK (renilla luciferase) vector was purchased from Promega (Madison, WI). The Nrf2 expression vector (pCMV6-Nrf2) was purchased from Origene (Rockville, MD) and pcDNA3.1 control vector was purchased from Invitrogen (Carlsbad, CA).

### Quantitative RT-PCR

After treatment, mRNA was isolated using Trizol reagent according to the manufacturer’s instructions (Invitrogen). The cDNA was prepared using the High Capacity Reverse Transcription kit from Applied Biosystems (Foster City, CA). The RT-PCR primers were synthesized at Midland Certified Reagent Company (Midland, TX) and SyBr Green Master Mix was purchased from Applied Biosystems (Foster City, CA). The following primer sequences were used for quantitative RT-PCR: *AR*: 5′-GGTGAGCAGAGTGCCCTATC-3′ and 5′-GAAGACCTTGCAGCTTCCAC-3′; *GAPDH*: 5′-TCCCATCACCATCTTCCA-3′ and 5′-CATCACGCCACAGTTTCC-3′; *PSA:* 5′-AGGCCTTCCCTGTACACCAA-3′ and 5′-GTCTTGGCCTGGTCATTTCC-3′; and *TMPRSS2*∶5′-GTCCCCACTGTCTACGAGGT-3′ and 5′-CAGACGACGGGGTTGGAAG-3′. Fold changes in gene expression were calculated after normalization to their corresponding GAPDH mRNA levels.

### Nuclear Protein Extraction

Nuclear pellets for westerns were isolated using the CER-I and CER-II extraction buffers from the NE-PER Nuclear Extraction kit (Pierce, Rockford, IL). Nuclei were washed with HBSS and nuclear protein was extracted with a custom nuclear protein lysis buffer [50 mM Tris-HCl (pH 7.4), 500 mM NaCl, 1% NP-40, 1% sodium-deoxycholate, 0.1% SDS, 1 mM phenylmethylsulfonyl fluoride (PMSF), and 1 mM EDTA]. Nuclear protein was quantified using the BCA protein estimation kit from Thermo Scientific (Rockford, IL).

### Immunoblotting

Protein samples were boiled in 1∶1 volume of protein and loading buffer [100 mM Tris (pH 6.8), 25% glycerol, 2% SDS, 0.01% bromophenol blue, 10% 2-mercaptoethanol] for five minutes. Nuclear proteins (20–50 µg) were electrophoresed on Tris-HCl PAGE gels and wet transferred to nitrocellulose membranes. After blocking with 5% milk in TBST buffer (TBS with 0.05% Tween-20), membranes were hybridized with the indicated antibodies. Bands were then detected using the Lumiglo chemiluminescent substrate system (KPL, Gaithersburg, MD). Band intensities were quantified with Image-J Software (NIH) and values were normalized to TBP protein levels in their respective samples.

### Transient Transfection and Luciferase Assays

Cells were seeded into 6-well plates overnight before transfection with Lipofectamine-2000 reagent (Invitrogen, Carlsbad, CA) and the indicated vectors. For luciferase assays, cells were transfected with either the psPSA-*luc* reporter plasmid alone or in combination with equal amounts of Nrf1 expression vector (p65-Nrf1-V5-His or p120-Nrf1-V5-His), or Nrf2 expression vector (pCMV6-Nrf2), or a control expression vector (pcDNA3.1). To normalize for transfection efficiency, cells were also cotransfected with the pRL-TK (renilla luciferase) vector. Western studies were also performed with the indicated expression vectors alone. In brief, cells were incubated overnight in transfection solution (20 µl Lipofectamine, 400 ng luciferase vector and/or expression vector, and 100 ng pRL-TK) in 2 ml of serum/phenol red free media. After overnight incubation, media was removed and cells were exposed to DHT (0, 1 or 10 nM) for 24 hrs in CS-FBS containing phenol red free RPMI. Cell extracts were harvested and luciferase levels were determined using the dual Luciferase Reporter Assay kit (Promega, Madison, WI). In each experiment, firefly luciferase values (from psPSA-luc) were normalized to renilla luciferase values (from pRL-TK). Nuclear protein was extracted after treatment and evaluated by western for changes in nuclear protein expression. In parallel experiments, cells were also transfected with the Nrf1 and Nrf2 expression vectors alone in order to monitor DHT-induced expression of two AR regulated genes, PSA (prostate specific antigen) and TMPRSS2 (transmembrane protease, serine 2).

### siRNA Transfection

Short interfering RNA (siRNA) transfections were carried out using Transfast reagent from Promega (Madison, WI). Briefly, cells were incubated overnight (∼18 hrs) in transfection solution that consisted of Transfast reagent (2∶1 dilution), and 20 nM of either Nrf1 siRNA or NC1 (control) siRNA in serum and phenol red free RPMI. For plasmid cotransfections, cells were simultaneously transfected with both siRNA and plasmid at a 2∶1 dilution of Transfast reagent, as described in the luciferase assay section. After overnight transfection, media was removed and cells were exposed to the indicated treatments. Cells were then harvested after 24 hrs and luciferase assays were carried out. In parallel samples, RNA and nuclear protein was obtained to monitor gene expression by qRT-PCR and by western.

### Immunoprecipitation

For co-immunoprecipitation and immunoblotting (co-IP/IB) studies, LNCaP and C4-2B cells were treated with 0, 1, or 10 nM DHT for 6 hrs. Nuclear pellets were isolated using a nuclear isolation buffer (10 mM HEPES, 10 mM KCl, 2 mM MgCl_2_, 100 µM EDTA, 500 µM DTT, 0.625% NP-40, protease inhibitor, and phosphatase inhibitors). After washing in PBS two times, RIPA buffer (150 mM NaCl, 10 mM Tris (pH 8.0), 5 mM EDTA, 1% Deoxycholate, 1% Triton X-100, 0.1% SDS, protease inhibitor, and phosphatase inhibitors) was added to nuclear pellets and pellets were sonicated to isolate nuclear protein. Protein was pre-cleared for 30 mins with protein-G/protein-A agarose beads (Calbiochem cat# IP10). AR antibody (Abcam; ab74272) was then added to 100 µg of nuclear protein in 400 µl RIPA lysis buffer and incubated at 4°C overnight. The protein-G/protein-A agarose beads were then added to protein and incubated for 2 hrs. After incubation, beads were washed 3 times in RIPA lysis buffer and loading dye was added to the sample. Samples were then boiled for 5 mins at 95°C, loaded onto the gel, and immunoblotted (IB) with the indicated antibodies.

### Chromatin Immunoprecipitation

For chromatin immunoprecipitation (ChIP) assays we used two different kits, the Covaris (Woburn, MA) truChIP chromatin shearing kit with non-ionic buffer and the Active Motif (Carlsbad, CA) ChIP-IT High Sensitivity kit. The assays were performed according to manufacturer’s protocols, with minor modifications. Briefly, DHT treated (6 hrs) LNCaP and C4-2B cells were sheared using the Covaris truChIP kit using an E220 focused-ultrasonicator from Covaris. The chromatin samples were diluted in ChIP buffer from the Active Motif kit. Samples were then immunoprecipitated (IP) with either the Nrf1 antibody (Proteintech Group) or the AR antibody (Active Motif). The remainder of the assay was performed according to the Active Motif kit instructions. ARE specific qRT-PCR was performed on the IP DNA using primers for ARE-II sequences located within the PSA-promoter (5′-CCACAAGATCTTTTT ATGATGACAG-3′ and 5′-GCTCATGGAGACTTCATCTAG-3′). Changes in amplified band intensities were quantified.

### Electrophoretic Mobility Shift Assays

The 2^nd^ generation DIG Gel Shift kit from Roche (Branford, CT) was used for EMSA studies. The androgen response element (ARE) and the TCF11 and TCF11/MafG (Nrf1 binding) sequences [29] were synthesized from the Midland Certified reagent company. The sequences for each EMSA oligonucleotide are as follows: *TCF11*∶5′-GTCATTT-3′ and 3′-AAATGAC-5′; *ARE*: 5′-GATCCTTGCAGAACAGCAA GTGCTAGCTG-3′ and 3′-GAACGTCTTGTCGTTCACGATCGACCTAG-5′; and *TCF11* ***/*** *MafG*: 5′-CCCAAATGACAATGCGATTGA-3′, 3′-TCAATCGCATTGTCATTTGGG-5′ (Genomatix MatInspector). Briefly, nuclear protein was extracted from cells treated with DHT for 6 hrs and binding reactions with DIG labeled ARE oligonucleotides were carried out. ARE oligos were incubated with nuclear protein (10 µg) for 30 min, after which sample loading buffer was added and samples were electrophoresed on a 5% TBE PAGE gel (Bio-Rad, Hercules, CA). Samples were then transferred onto a nylon membrane, incubated with blocking buffer, exposed to an anti-DIG antibody, washed, and developed using the ECL chemiluminescent system, as described previously. For competition studies, nuclear extracts were pre-incubated with excess (50-fold) unlabeled ARE, TCF11, or TCF11/MafG oligos for 30 min before DIG-labeled ARE oligos were added to the reaction. For experiments using antibodies, to compete for Nrf1 binding, nuclear extracts were pre-incubated with either Nrf1 antibody or with rabbit IgG (non-specific control) for 30 min before DIG-labeled ARE oligos were added to the reaction. Electrophoresis, transfer, and hybridization were carried out as described above.

### Statistical Analysis

Each treatment condition consisted of 2–4 replicates and each experiment was performed 2-5 times. Relative expression was determined by comparing treatment values to control values after normalization to loading controls. Statistical significance was evaluated by two-way ANOVA using the GraphPad Prism software. Significant changes from controls are indicated by p-values of <0.05.

## Results

### AR Transactivation Levels are Significantly Higher in C4-2B Cells than in LNCaP Cells

**Figure 1:**
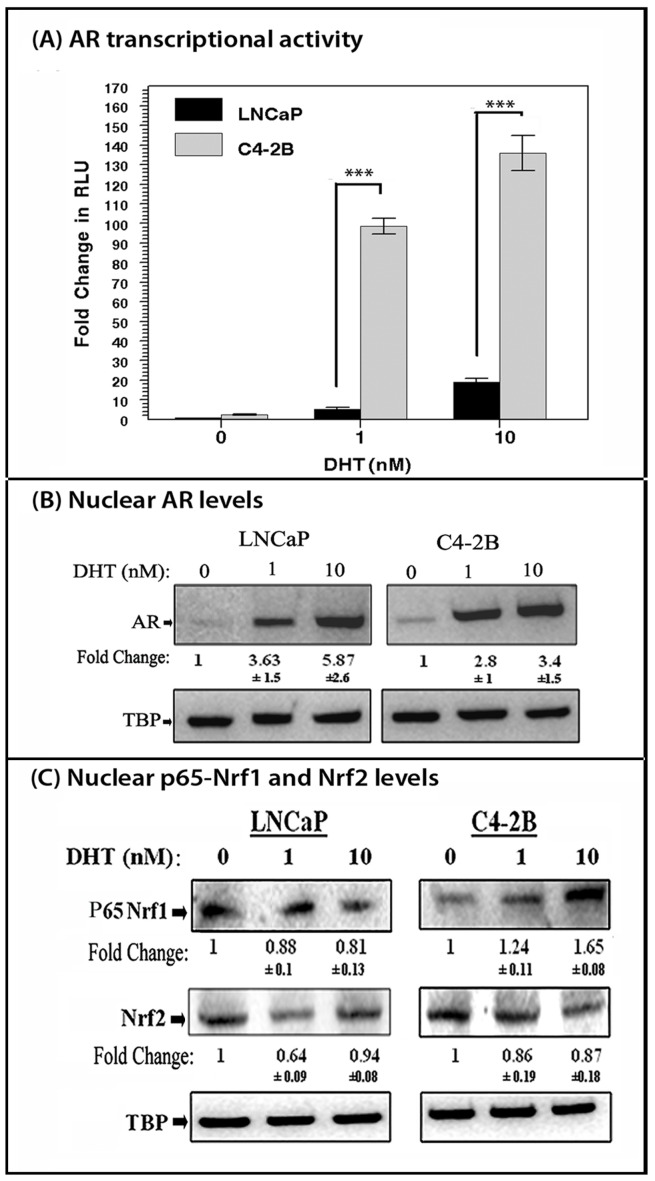
Differential effects of DHT in LNCaP and C4-2B cells: AR transactivation, and nuclear AR, p65-Nrf1 & Nrf2 levels. (A). Cells were cotransfected with psPSA-*luc* and pRL-TK (internal control). The effect of 24 hrs stimulation with either 1 nM or 10 nM DHT on fold changes in luciferase activity (firefly/renilla RLU) are shown (n = 3). DHT-induced AR transactivation is significantly (***; p<0.001) higher in C4-2B cells as compared to LNCaP cells. (B). DHT-induced AR nuclear localization in LNCaP and C4-2B cells. Following 24 hrs of DHT (0, 1 and 10 nM) stimulation (n = 4) western immunoblots show changes in AR nuclear levels. Both C4-2B and LNCaP cells showed similar levels of nuclear AR following DHT-stimulation. (C). p65-Nrf1 and Nrf2 levels following DHT stimulation. Western immunoblots showing nuclear p65-Nrf1 and Nrf2 levels following 24 hr of DHT stimulation (n = 3). Differences in nuclear p65-Nrf1 and Nrf2 were observed in LNCaP and C4-2B cells. Fold changes and ±SEM values represent relative differences in the expression of AR, p65-Nrf1, and Nrf2. In both (B) and (C), data were normalized to TBP levels in each sample.

To compare DHT-induced AR transactivation levels in LNCaP and C4-2B cells, we carried out luciferase assays in psPSA-*luc* vector transfected cells (Fig. 1A). We observed that AR transactivation was significantly (p<0.001) higher in C4-2B cells than in LNCaP cells. Following DHT treatment, LNCaP cells showed a dose dependent increase in AR transactivation (5 fold at 1 nM and 21 fold at 10 nM). In contrast, in C4-2B cells, a 100 fold increase was observed even at 1 nM DHT (p<0.001) and a more than 130-fold increase (p<0.001) was seen following exposure to 10 nM DHT (Fig. 1A). We also carried out immunoblotting studies to determine nuclear AR levels under both unstimulated and DHT-stimulated conditions (Fig. 1B). In both cell lines, a 2-5 fold increase in nuclear AR levels was seen after 24 hrs DHT-stimulation. Despite having similar nuclear AR levels after DHT treatment, C4-2B cells showed significantly higher AR transactivation levels as compared to LNCaP cells. This indicated that additional mechanisms that potentiate DHT-stimulated AR transactivation are present in the C4-2B cells.

### DHT Mediated Regulation of p65-Nrf1 and Nrf2 Nuclear Localization

We first determined if DHT treatment changes Nrf1 and Nrf2 nuclear localization (Fig. 1C). Nuclear levels of p65-Nrf1 were differentially affected in LNCaP and C4-2B cells. In C4-2B cells, DHT stimulation increased nuclear p65-Nrf1 levels, while in LNCaP cells DHT-stimulation did not significantly change nuclear p65-Nrf1 levels. However, Nrf2 nuclear localization was not significantly modified by DHT treatment in either LNCaP or C4-2B cells (Fig. 1C). Therefore, we investigated the ability of p65-Nrf1 and Nrf2 to regulate AR transactivation in both PCa cell lines.

### Modulation of p65-Nrf1 Expression Significantly Altered DHT-induced AR Transactivation

**Figure 2:**
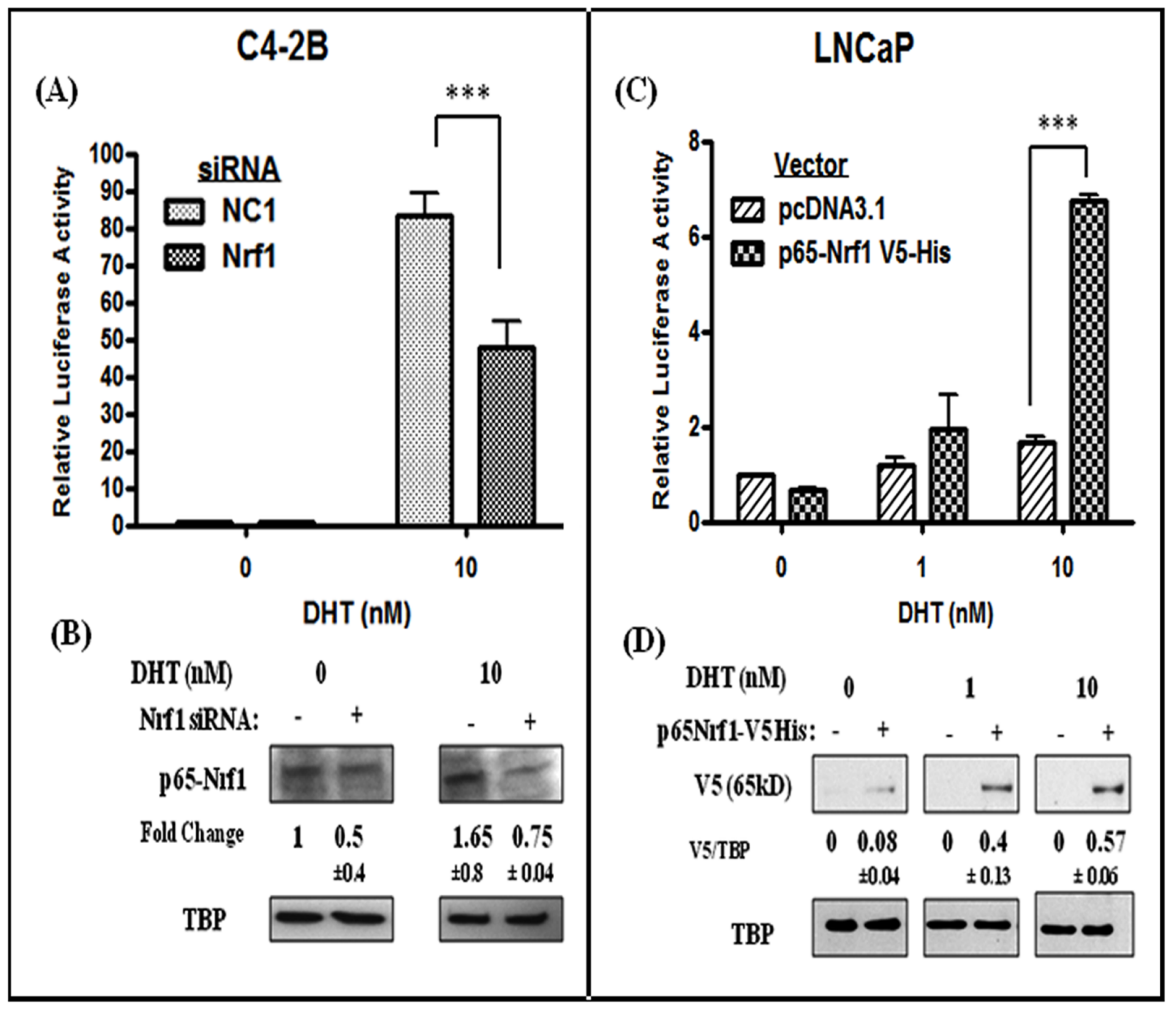
Effect of Nrf1 modulation on DHT-induced AR transactivation. (A) Effect of Nrf1 knockdown on AR transactivation in DHT-stimulated C4-2B cells. Cells were co-transfected with the psPSA-luc vector and with either the Nrf1 siRNA or control siRNA (NC1). Fold changes in luciferase activity (firefly/renilla RLU) following Nrf1 knockdown are shown (n = 3; p<0.01). (B) Nuclear p65-Nrf1 protein levels after siRNA mediated knockdown in C4-2B cells (n = 2). Data were normalized to TBP levels. (C) Effect of p65-Nrf1 overexpression on DHT-induced AR transactivation in LNCaP cells. Cells were co-transfected with the psPSA-luc vector and with either the control vector (pcDNA3.1) or the p65-Nrf1 expression vector (p65-Nrf1-V5-His). Fold change in luciferase activity following Nrf1 overexpression are shown (n = 3; p<0.001). (D) Changes in nuclear V5 protein (tag) in pcDNA3.1 (control) or p65-Nrf1-V5-His transfected LNCaP cells (n = 2). Fold changes represent relative (V5/TBP) differences in Nrf1.

We examined whether ectopic changes in Nrf1 levels can affect AR transactivation in DHT-stimulated LNCaP and C4-2B cells (Fig. 2). Since nuclear p65-Nrf1 is constitutively higher in C4-2B cells than in LNCaP cells [36], we used siRNA to silence endogenous Nrf1 levels in C4-2B cells (Fig. 2A & 2B) and a V5-His driven p65-Nrf1 expression vector (p65Nrf1-V5-His) to overexpress p65-Nrf1 in LNCaP cells(Fig. 2C & 2D). The levels of nuclear Nrf1 in siRNA transfected C4-2B cells and the levels of V5 fusion proteins in Nrf1 overexpressed LNCaP cells are shown in figures 2B and 2D, respectively. In C4-2B cells cotransfected with the psPSA-*luc* vector, and with either Nrf1 siRNA or NC1 control siRNA, luciferase assays showed that Nrf1 suppression significantly (p<0.001) reduces AR transactivation (Fig. 2A). In LNCaP cells, luciferase assays also showed that p65-Nrf1 overexpression enhances DHT-stimulated AR activity by approximately 2-fold at 1 nM DHT and by approximately 4.4-fold at 10 nM DHT (p<0.001) (Fig. 2C). These findings suggested that p65-Nrf1 enhances AR transactivation AR transactivation in both PCa cell lines.

In parallel studies, we also measured the expression of two AR regulated genes, PSA and TMPRSS2, in cells transfected with the p65-Nrf1 expression vector (Fig. S1-A & S1-B). Our qRT-PCR data clearly showed that p65-Nrf1 significantly (p<0.01) increases both TMPRSS2 and PSA mRNA levels in DHT-treated LNCaP cells. This served as a corroborative evidence that p65-Nrf1 is a potential AR coactivator.

### Nrf2 Negatively Regulates AR Transactivation

**Figure 3:**
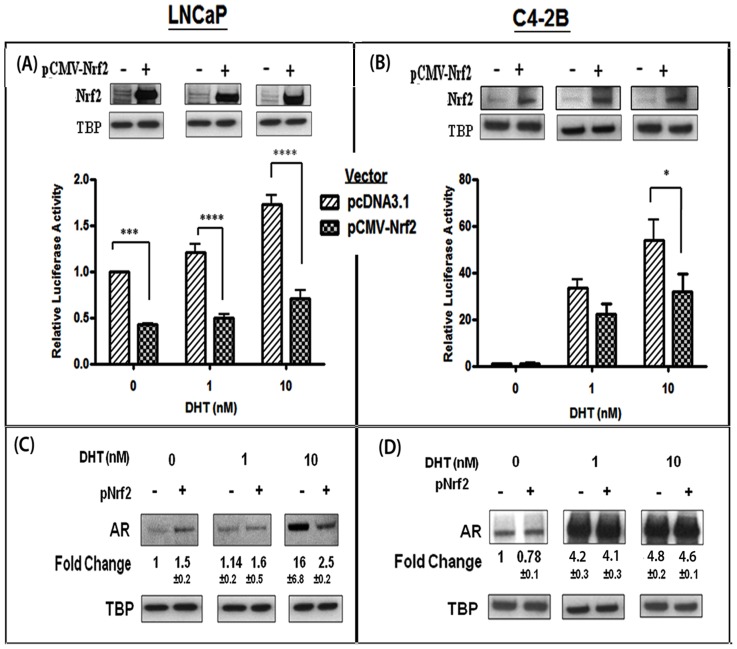
Effect of Nrf2 on DHT-induced AR nuclear localization and AR transactivation. The effects of Nrf2 overexpression on DHT-stimulated AR activity were monitored in both LNCaP (A) and C4-2B (B) cells. Cells were transfected with the psPSA-luc reporter plasmid, and with either the Nrf2 expression vector (pCMV-Nrf2) or the control vector (pcDNA3.1) and stimulated with DHT (0–10 nM) for 24 hrs (n = 5). Significant differences in luciferase activity (firefly/renilla RLU) from controls are represented as *; p<0.05, ***; p<0.001 and ****; p<0.0001. In both (A) and (B), panels above the bar graphs represent changes in nuclear Nrf2 levels after transfection with pCMV-Nrf2. In (C) and (D), effects of Nrf2 overexpression on nuclear levels of AR in both untreated and DHT (0, 1, 10 nM) treated LNCaP (C) and C4-2B (D) cells are shown. Data were normalized to nuclear TBP levels. Fold changes and ±SEM values represent differences in nuclear AR levels as compared to untreated cells.

The effects of Nrf2 overexpression on AR transactivation were monitored in both LNCaP and C4-2B cells (Fig. 3). Nrf2 overexpression significantly suppressed DHT-stimulated AR activity. In LNCaP cells (Fig. 3A), Nrf2 overexpression suppressed AR transactivation under both basal (50%; p<0.001) and DHT-stimulated conditions (60%; p<0.0001). Nrf2 overexpression also reduced DHT-induced AR transactivation levels in C4-2B cells (Fig. 3B) by approximately 33% at 1 nM DHT and by 40% at 10 nM DHT (p<0.05). Nuclear Nrf2 expression in transfected LNCaP and C4-2B cells are shown above each treatment conditions.

Next, the effect of Nrf2 overexpression on nuclear AR levels were measured in both LNCaP cells (Fig. 3C) and C4-2B cells (Fig. 3D). Interestingly, in DHT-treated LNCaP cells, Nrf2 overexpression reduced AR nuclear localization by almost 79%. However, Nrf2 overexpression did not significantly alter nuclear AR levels in DHT-treated C4-2B cells. These findings indicate that Nrf2 may be a potent negative regulator of DHT-induced AR transactivation in both PCa cell lines. However, in LNCaP cells, but not in C4-2B cells, this suppressive effect may be mediated via suppression of AR nuclear localization.

### Changes in Nrf1 and Nrf2 do not Alter AR Gene Expression or AR Nuclear Localization

We next evaluated whether modifications in Nrf1 and Nrf2 overexpression affect AR gene expression and AR nuclear localization (Fig. S2). qRT-PCR studies showed that neither p65-Nrf1 overexpression in LNCaP cells (Fig. S2-A) nor p65-Nrf1 knockdown in C4-2B cells (Fig. S2-B) altered AR mRNA levels, under either basal or DHT-stimulated conditions. Western immunodetection studies showed that DHT-induced nuclear localization of AR was unaffected by either p65-Nrf1 overexpression (Fig. S2-C) or Nrf1 knockdown (Fig. S2-D). Similarly, Nrf2 overexpression in DHT-treated LNCaP and C4-2B cells did not significantly affect AR gene expression (Fig. S2-E & S2-F). Thus, Nrf1 does not modify AR transactivation through regulation of either AR gene expression or AR nuclear localization. Furthermore, the suppressive effects of Nrf2 are not due to alterations in AR gene expression.

### Protein-protein Interactions between Nuclear Nrf1 and AR Occur in both LNCaP and C4-2B Cells

**Figure 4:**
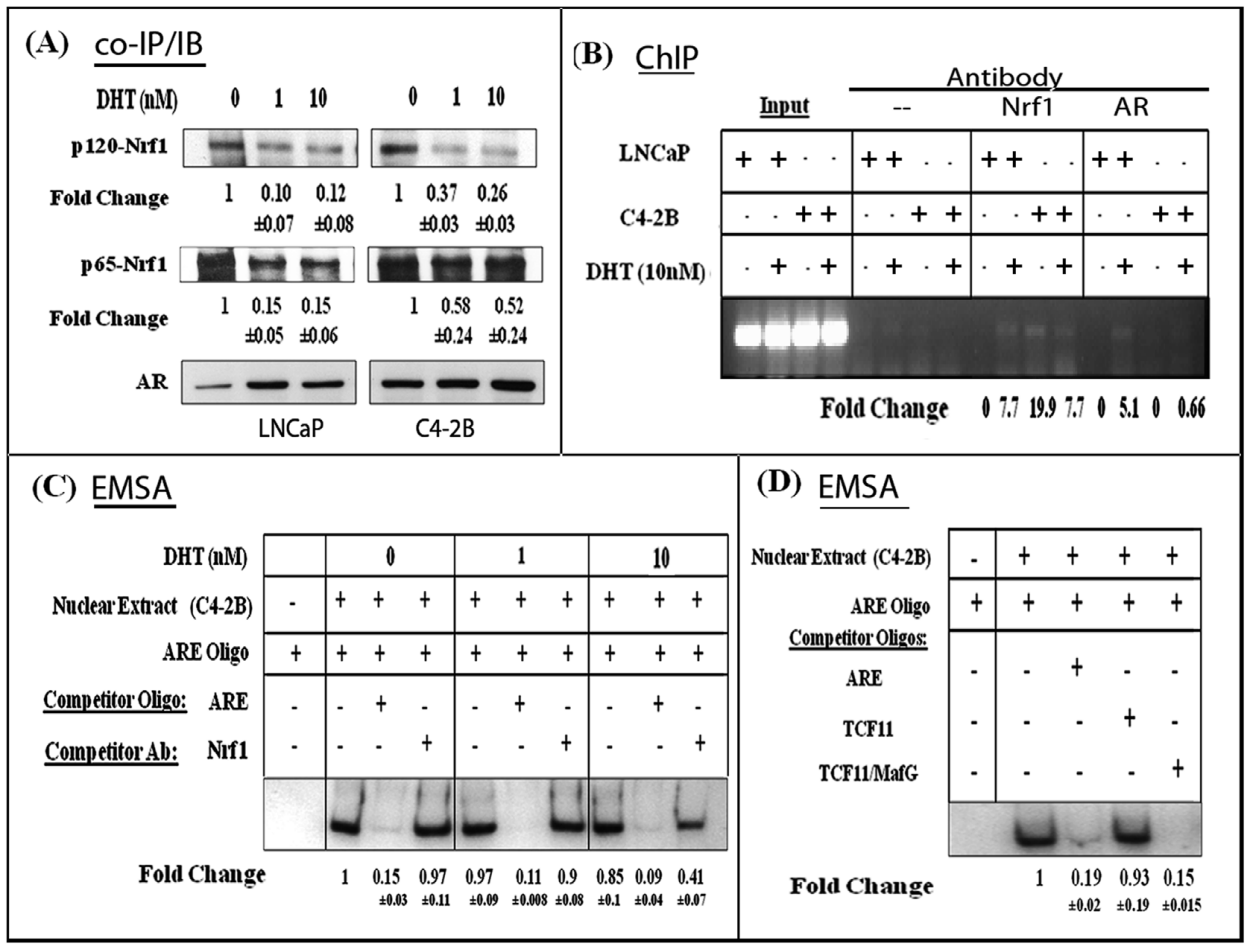
Nrf1 association with the AR transactivation complex at the ARE. (A) Co-IP/IB studies of Nrf1 binding to AR in nuclear extracts from DHT-stimulated cells. AR was immunoprecipitated (IP) from nuclear extracts of LNCaP and C4-2B cells, electrophoresed on SDS-PAGE and immunoblotted (IB) with Nrf1 antibodies. Changes in p120-Nrf1 (120 kDa) and p65-Nrf1 (65 kDa) interaction with nuclear AR are shown (n = 3). In the bottom lane, nuclear AR levels in DHT-stimulated cells are shown as a positive control. Data were normalized to nuclear TBP levels (not shown) and fold change and ±SEM values represent relative differences in Nrf1 proteins. (B) AR and Nrf1 interactions at the androgen response element. ChIP assays were carried out using nuclear extracts from DHT treated LNCaP and C4-2B cells. Protected chromatin regions were amplified using ARE specific PCR primers. The ‘input’ represents PCR products amplified directly from the sheared chromatin. Products generated following no antibody incubation was used as a negative control, and those generated following incubation with either the anti-AR or anti-Nrf1 antibody were used to show Nrf1-AR interactions at the ARE sequences. A representative showing the ARE PCR products from each treatment group is shown. In (C) & (D), Nrf1-AR interaction with the labeled ARE was monitored by EMSA and specificity of binding established with competition with either antibodies or oligonucleotides. (C) Nuclear extracts from DHT-treated C4-2B cells were pre-incubated with Nrf1 antibody before addition of the labeled ARE oligo. (D) Competition with excess (50-fold) of unlabeled ARE oligos, unlabeled TCF11, or unlabeled TCF11/MafG (Nrf1 specific) oligos (n = 2). In each experiment, fold changes represent relative differences in protein complex formation at the ARE.

To determine if the Nrf1 proteins (p65- and p120-) regulate AR function via direct interaction with nuclear AR protein, we performed AR immunoprecipitations (IP) from nuclear extracts of untreated and DHT-treated LNCaP and C4-2B cells (Fig. 4A). The IP proteins were then immunoblotted (IB) for either p65-Nrf1 or p120-Nrf1. The Co-IP/IB studies showed that both p65-Nrf1 and p120-Nrf1 associate with nuclear AR protein in both LNCaP and C4-2B cells. However, nuclear AR interacted with p65-Nrf1 at a much higher level than that observed with p120-Nrf1. Furthermore, AR interaction with p120-Nrf1 was significantly reduced within 6 hours of DHT-stimulation and the decrease in p65-Nrf1 interactions with AR was more prominent in nuclear extracts from DHT-treated LNCaP cells than in C4-2B cells. This suggests that both Nrf1 isoforms (p65 and p120) can interact with AR, but their interaction is differentially regulated in androgen dependent and castration resistant PCa cells. Modified levels of p65-Nrf1 and p120-Nrf1 interaction with nuclear AR in C4-2B cells, as compared to LNCaP cells, indicates that Nrf1 may be involved in the induction of AR transactivation in CRPC cells.

### Nrf1 Associates with AR Transcription Complexes Formed at the ARE

To determine whether the Nrf1 and AR interactions in C4-2B cells can facilitate transcription factor complex formation at the ARE sequences, we performed both ChIP assays (Fig. 4B) and EMSAs (Fig. 4C & 4D) using nuclear extracts from DHT (10 nM) treated LNCaP and C4-2B cells. We used primers specific for the ARE element of the PSA promoter. In Figure-4B, the first four lanes indicate the input (positive controls) which represents PCR products amplified directly from total chromatin from each treatment group. In the second four lanes, we used a no antibody control as a negative control. In the following lanes, the indicated PCR products were generated following incubation with either the Nrf1 antibody or the AR antibody, which showed both proteins interacting at the ARE in the PSA promoter. Chip assays with the Nrf1 antibody indicate that the binding of Nrf1 to the AR complex at the ARE is DHT dependent. However, in C4-2B cells, Nrf1 is present at the ARE when there is no hormone present, suggesting that C4-2B cells have an increased capacity to use Nrf1 to enhance ARE mediated transcription. Therefore, although Nrf1 binding to the AR transcription complex requires DHT-stimulation of the LNCaP cells, Nrf1 is constitutively bound to the AR transcription complex in C4-2B cells. This suggested that C4-2B cells have an increased capacity to use Nrf1 to enhance ARE directed transcription. It is important to note that since we used an antibody that recognizes both Nrf1 isoforms, our ChIP data does not discriminate between the binding of either p65-Nrf1 or p120-Nrf1. In addition, our chip assay data demonstrating Nrf1 differentially binding at the ARE in the presence of hormone correlates with our IP studies that showed differential responses to hormone in LNCaP cells and C4-2B cells (Fig. 4A).

Next, we conducted EMSA studies to further corroborate Nrf1 association with the AR transcription complex at the ARE, using a DIG labeled ARE oligonucleotide and nuclear extracts from DHT treated C4-2B cells (Figs. 4C & 4D). In all EMSA experiments, control reactions containing labeled ARE oligos, nuclear extracts, and competition with excess (50-fold) unlabeled ARE oligo, were performed to ascertain the specificity of the band. Transcription factor binding to the ARE was strongly competed out by preincubation with excess unlabeled ARE oligos. To examine the effects of Nrf1 on AR protein binding to ARE oligos, Nrf1 antibody was preincubated with the nuclear extracts before addition of labeled ARE oligos. Preincubation with the Nrf1 specific antibody significantly reduced the intensity of the complexes formed at the ARE (Fig. 4C). However, no reduction in ARE binding was observed upon preincubation with a non-specific rabbit immunoglobulin (IgG) (data not shown). Nrf1 binds to the TCF11/MafG site with high affinity as a heterodimer with MafG or as a homodimer; however, the TCF11 half site exhibits limited sequence specificity [29]. Therefore, to further investigate the specificity of Nrf1 binding to the ARE complex, we performed additional competition studies by preincubating the nuclear extracts with excess (50X) unlabeled oligonucleotides towards TCF11/MafG (Nrf1 binding site) or the half site (TCF11) alone (Fig. 4D). Indeed, competition with excess TCF11/MafG oligos, but not with the TCF11 oligos, significantly reduced complex formation at the ARE. Therefore, the co-IP/IB, ChIP and EMSA data suggest that Nrf1 associates with AR transcription complexes formed at the ARE (Fig. 4, A–D). The inductive effects of p65-Nrf1 on AR transactivation in DHT-treated C4-2B cells may thus be mediated via its direct interactions with AR at the ARE.

### Nrf2 Mediated Downregulation of AR Function Occurs via the Modulation of p120-Nrf1 Levels

**Figure 5:**
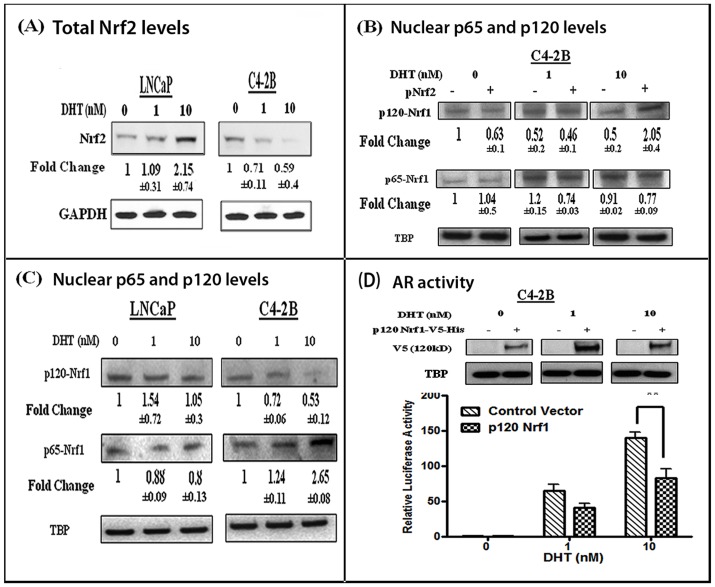
Role of p120-Nrf1 in Nrf2 mediated repression of AR transactivation. (A) Effect of DHT on total Nrf2 protein levels in LNCaP and C4-2B cells (n = 3). Data were normalized to GAPDH protein levels in respective samples. Fold changes and ±SEM values represent relative differences in the expression of Nrf2. (B) Effect of Nrf2 on nuclear p65-Nrf1 and p120-Nrf1 levels in C4-2B cells. Cells were transfected with either pcDNA3.1 or pCMV-Nrf2 and stimulated with DHT (0–10 nM) for 24 hrs. Nuclear extracts were evaluated for changes in nuclear p65-Nrf1 and p120-Nrf1 protein levels (n = 2). (C) Effect of DHT treatment on nuclear protein levels of both p65-Nrf1 and p120-Nrf1 in LNCaP and C4-2B cells. In both (B) and (C), densitometric values for each band was normalized to TBP levels and fold changes compared to control (0 nM DHT) are shown (n = 3). (D) Effect of p120-Nrf1 overexpression on DHT-induced AR activity in C4-2B cells. Cells were cotransfected with the psPSA-luc vector and with either pcDNA-3.1 (control) or the p120-Nrf1 expression vector (p120-Nrf1-V5-His) and exposed to DHT (0–10 nM) for 24 hrs and luciferase assays were performed using whole cell extracts. Significant differences in luciferase activity (firefly/renilla RLU) from controls are represented as **; p<0.01 (n = 3). Panels above each bar graph show nuclear V5-His expression in cells transfected with either control or p120-Nrf1 expression vectors (n = 2).

Next, we wanted to investigate the mechanism/s via which Nrf2 down- regulates AR transactivation, as shown in Fig. 3. Therefore, we initially performed co-IP/IB studies to determine whether Nrf2 directly associates with AR in nuclear extracts from control and DHT-treated cells. Unlike that observed with Nrf1 (Fig. 4), we did not observe any interactions between AR and Nrf2, in either LNCaP or C4-2B cells (data not shown). However, we observed that total Nrf2 protein levels were differentially regulated in DHT-treated LNCaP and C4-2B cells (Fig. 5A). In contrast to nuclear Nrf2 levels (Fig. 1), total Nrf2 levels were increased (>2-fold) in DHT-stimulated LNCaP cells and decreased to 40% in DHT-stimulated C4-2B cells. Therefore, we subsequently investigated if Nrf2 has a direct effect on Nrf1 expression or its nuclear localization in the C4-2B cells. We observed that while Nrf2 overexpression does not affect the nuclear localization of p65-Nrf1, it significantly enhances (>2 fold) nuclear p120-Nrf1 levels in C4-2B cells (Fig. 5B), but not in LNCaP cells (data not shown). These findings indicate that the inhibitory effects of Nrf2 on AR signaling may be manifested via increased p120-Nrf1 nuclear levels, which may compete with p65-Nrf1 for binding to AR. Indeed, as shown in Fig. 5C, we observed that DHT-stimulation specifically increased (>2.5 fold) nuclear p65-Nrf1 and decreased (>50%) nuclear p120-Nrf1 in C4-2B cells. Interestingly, however, this differential effect of DHT on Nrf2 nuclear localization was not seen in either LNCaP cells or C4-2B cells. Therefore, we monitored whether p120-Nrf1 can directly affect AR transactivation using psPSA-luc transfected cells. The inhibitory effect of p120-Nrf1 on AR transactivation was demonstrated by experiments showing that overexpression of p120-Nrf1 reduces AR transactivation in DHT treated C4-2B cells (Fig. 5D). These data suggest that reciprocal changes in nuclear levels of both p65-Nrf1 and p120-Nrf1 may differentially alter AR transactivation function in LNCaP and C4-2B cell lines.

## Discussion

Despite an initial response to hormone deprivation, most PCa patients relapse to a hormone refractory state in which tumors utilize enhanced AR function to survive during ADT. Several studies have indicated that this may be the result of augmented AR transactivation in CRPC cells [14], [15], [17]. Our previous findings demonstrated that NOX-4 and NOX-5, Prx-1, and the oxidative stress-induced transcription factors Nrf1 and Nrf2 are differentially expressed in LNCaP (androgen dependent) and C4-2B (castration resistant) cells [36]. Our current studies show that the two Nrf1 isoforms (p65-Nrf1 and p120-Nrf1) and Nrf2 are modulated by DHT and that they differentially affect AR transactivation in hormone dependent and hormone independent PCa cells.

**Figure 6:**
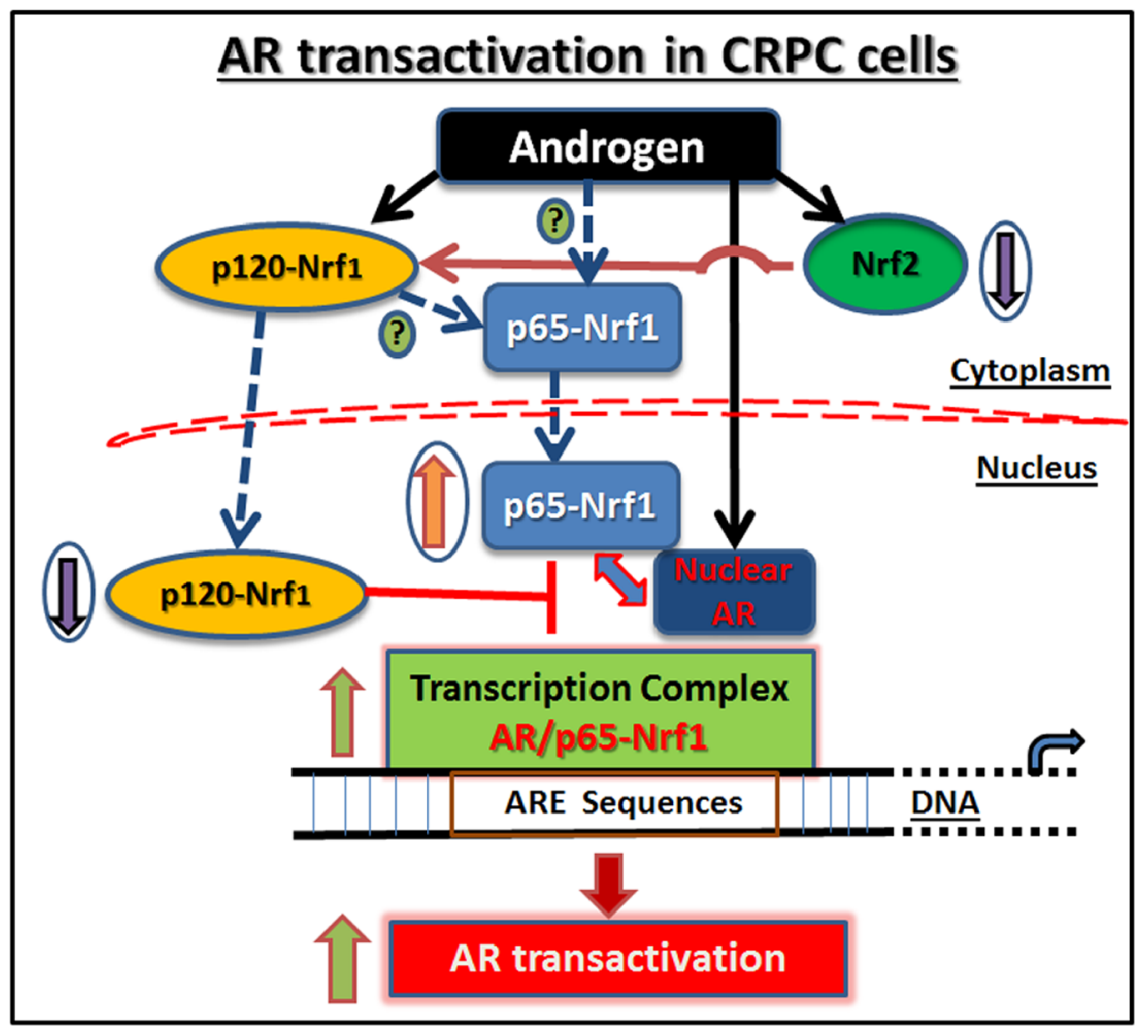
Mechanism of AR transactivation in CRPC cells: role of Nrf2, p65-Nrf1 and p120-Nrf1. In CRPC cells (e.g. C4-2B), despite ADT, residual androgens (e.g. DHT) increase nuclear p65-Nrf1 and simultaneously decrease both total Nrf2 and nuclear p120-Nrf1 levels. Nuclear p65-Nrf1 associates with nuclear AR and with the AR transcription complex bound to the ARE sequences in promoter/enhancer regions of genes that regulate prostate tumor growth. Therefore, CRPC cells may utilize the AR coactivator p65-Nrf1 to enhance AR transactivation and facilitate the recurrence of PCa.

We present a novel mechanism in which oxidative stress-induced transcription factors are utilized by CRPC cells to increase AR function despite low hormone levels during ADT. The levels of Nrf2 and the ratio of p65-Nrf1 to p120-Nrf1 in PCa cells dictate their effects on the activity of AR. Differential regulation of the interactions that occur between these proteins and the AR transcription complex may dictate AR function in both hormone dependent LNCaP cells and hormone independent C4-2B cells. Here, we present a schematic model of the differential actions of Nrf1 and Nrf2 in regulating AR transactivation function in CRPC cells (Fig. 6).

Compared to LNCaP cells, DHT-stimulated C4-2B cells showed elevated nuclear p65-Nrf1 levels which were associated with enhanced AR transactivation in these CRPC cells. Indeed, compared to LNCaP cells, psPSA-luc transfected C4-2B cells had a significantly greater capacity for AR transactivation at a reduced DHT concentration (1nM), even though DHT induced patterns of AR nuclear localization were similar in both cell lines (Fig. 1B). DHT stimulation modified p65-Nrf1 nuclear levels (Fig. 1C) and ectopic modulation of p65-Nrf1 enhanced AR transactivation in both LNCaP and C4-2B cells (Fig. 2) which indicated that C4-2B cells utilize p65-Nrf1 as a potential AR coactivator. This indicates that androgen independent C4-2B cells utilize additional mechanisms to enhance hormone sensitivity that are not present in LNCaP cells.

Mechanistic studies suggested that p65-Nrf1 mediates its inductive effects on AR transactivation by directly interacting with the AR transcription complex (Fig. 4) and that p120-Nrf1 inhibits the effects of p65-Nrf1 on AR signaling (Fig. 5). Although the co- immunoprecipitation studies indicated that Nrf1 interactions with nuclear AR are decreased in the presence of DHT, this reduction was greater in LNCaP cells than in C4-2B cells (Fig. 5A) and the suppression of p120-Nrf1/AR interactions by DHT was greater than the suppression of p65-Nrf1/AR interactions (Fig. 5B). Furthermore, the pronounced suppressive effect of p120-Nrf1 also suggested that AR activation in C4-2B cells may be facilitated by a simultaneous decrease in the inhibitory effects of p120-Nrf1; thus further increasing the stimulatory effects of augmented p65-Nrf1. Indeed, both ChIP (Fig. 4B) and EMSA studies (Fig. 4C & 4D) showed that the Nrf1-AR interactions occur at the ARE, especially in the presence of DHT. Competition for binding to the AR transcriptional complex using an Nrf1 antibody or the Nrf1 binding oligonucleotide TCF11/MafG, showed that Nrf1 is present in the AR transcriptional complex at the ARE. Studies with the two Nrf1 isoforms showed that, AR transactivation is enhanced in the presence of elevated p65-Nrf1 (Fig. 2) and reduced when p120-Nrf1 is overexpressed (Fig. 5). Since the NTD of p120-Nrf1 can bind to nuclear and ER membranes, it is possible that p120-Nrf1 negatively regulates AR transactivation through sequestration of AR away from the nuclear DNA [28]. Further investigation will be required to determine the specific mechanism/s by which p120-Nrf1 regulates AR function.

In contrast to p65-Nrf1, the suppressive effect of Nrf2 on AR transactivation was seen in both LNCaP and C4-2B cells (Fig. 3). Nrf2 overexpression significantly reduced AR transactivation and differentially regulated AR nuclear localization. Although the DHT-induced nuclear localization of Nrf2 was not significantly different in either cell line (Fig. 1), total Nrf2 expression was decreased within 6 hrs of DHT treatment in C4-2B cells, and it’s total expression increased in response to DHT in LNCaP cells (Fig. 5A). This implicates a role for Nrf2/AR interactions in regulating AR transactivation. However, our co-IP/IB and competition studies indicated that Nrf2 does not directly interact with nuclear AR (data not shown). Therefore, the suppressive effect of Nrf2 on AR transactivation may be mediated indirectly. Indeed, we observed that overexpression of Nrf2 significantly enhanced the nuclear localization of p120-Nrf1 in C4-2B cells (Fig. 5) but not in LNCaP cells (data not shown). Further investigations revealed that p120-Nrf1 overexpression in C4-2B cells can also reduce AR transactivation (Fig. 5). Since DHT stimulation decreased the nuclear levels of p120-Nrf1 while increasing the nuclear levels of p65-Nrf1 in C4-2B cells, our findings suggest that Nrf2 reduces AR transactivation by increasing nuclear p120-Nrf1 levels. Our findings imply that under physiological settings of ADT, a long-term shift in the Nrf1 and Nrf2 balance in cancer cells may result in enhanced AR function in a subpopulation of cells and enable the outgrowth of CRPC.

Previous studies suggested multiple roles for Nrf2 in cancer progression, recurrence, and resistance [25]. Increased expression of Nrf2, and its downstream antioxidant genes, can provide cancer cells with a survival advantage via their protection from oxidative stress [40]. However, growth of some tumors are enhanced by Nrf2 knockdown [41] and decreased Nrf2 levels correlate with increased PCa tumor grade [33]. Reduced Nrf2 expression has also been linked to increased prostate tumorigenesis in a mouse model of PCa [32]. Our molecular findings suggest that the CRPC cells may bypass the suppressive effects of Nrf2 on AR transactivation during ADT by selecting for tumor clones with decreased Nrf2 expression. This could be a novel therapeutic approach to target CRPCs. Since Nrf2 expression in adults decreases with age [42] and PCa incidence is higher in aging men [1], our studies also implicate an association between decreased Nrf2 expression and more aggressive PCa in the elderly [32], [33].

Despite its potential role in regulating Nrf2 expression or its ability to regulate Nrf2 function, little is known about the role of Nrf1, or its isoforms, in regulating PCa progression [30], [35]. In LNCaP and C4-2B cells, the differential regulation of p65-Nrf1 and p120-Nrf1 nuclear localization by DHT clearly indicated that Nrf1 expression may change during PCa progression, especially in CRPC cells that have reduced Nrf2 levels [36]. The ability of Nrf2 to enhance p120-Nrf1 nuclear localization further suggests that any decrease in total Nrf2 expression would result in relieving the inhibition of AR signaling by p120-Nrf1. Therefore, if the effect of Nrf1 on AR signaling is dependent upon the ratio of ratio of p65-Nrf1 to p120-Nrf1 and their binding to the AR transcription complex, reducing the expression of Nrf2 would reduce nuclear p120-Nrf1 expression and reduce the competition for p65-Nrf1 binding to AR. Further investigation will be required to elucidate how Nrf2 expression is differentially regulated by hormone in both LNCaP and C4-2B cells, how Nrf2 regulates p120-Nrf1 nuclear localization. In addition, the mechanisms that regulate the interchangeable association of p65-Nrf1 and p120-Nrf1 with the AR transcription complex at the ARE will need to be properly understood. A clear understanding of how DHT regulates the processes that mediate p65-Nrf1, p120-Nrf1, and Nrf2 localization, expression and activity, could provide novel strategies for targeting persistent AR signaling in aggressive CRPC cells.

One therapeutic strategy could be to target the proteosomal pathway that may regulate p65-Nrf1 processing and its subcellular localization. Chepelev et al. (2011), recently described several mechanisms by which the full length Nrf1 (p120-Nrf1) may be processed into its smaller isoforms via the 26S proteasome pathway [35]. The proteasomal inhibitor, MG-132 can stabilize the expression of full-length Nrf1. Interestingly, the proteasome pathway is known to regulate AR activity in PCa cells [43] and Celastrol, a potent proteasome inhibitor, can suppress growth of PCa xenografts in nude mice [44]. Since the NTD of p120-Nrf1 is known to facilitate its integration into the ER and nuclear membranes [27], [38]. Strategies to suppress this nuclear export signal in the NTD of p120-Nrf1 could increase its nuclear localization and reduce AR activity in CRPC cells [45].

A third strategy may be to target the degradation of p120-Nrf1 directly. Steffen et al. (2010), have shown that the violin-containing protein (VCP/p97), a ubiquitin proteasome associated protein required to extract ubiquitylated proteins from membranes before their proteasomal degradation, can facilitate p120-Nrf1 degradation [46] **.** Indeed, p120-Nrf1 levels were found to be significantly increased in cells following inhibition of VCP expression. Interestingly, elevated VCP expression has been previously associated with poor prognosis in PCa patients [47]. Patients whose VCP levels were elevated had increased serum PSA levels and higher Gleason scores. Furthermore, reversible inhibitors of VCP, such as N^2^, N^4^-dibenzylquinazoline-2,4-diamine (DBeQ), have been shown to potently inhibit PCa cell growth [48]. Thus, compounds that can modulate p120-Nrf1 processing and/or increased nuclear localization may reduce AR activity in CRPC cells. A fourth strategy may be to augment the inhibitory effects of Nrf2 on AR transactivation by upregulating Nrf2 expression in PCa cells. Nrf2 inducers may act to reduce AR activity in CRPC cells [49]. Indeed, sulforaphane, an active component of broccoli sprouts, can increase Nrf2 levels and has demonstrated potent anti-cancer activity in the TRAMP PCa mouse model [50]. In addition, the curcumin analog 27 (ca27), which potently activates Nrf2, was also shown to downregulate AR expression and function in several PCa cell lines [51]. Therefore, strategies to augment Nrf2 levels may be used to treat advanced prostate cancer and prevent their progression to CRPC.

In summary, our current investigations demonstrate new roles for Nrf1 and Nrf2 in regulating AR signaling in PCa. A clearer understanding of the opposing relationships between the various Nrf1 isoforms (p65-Nrf1 and p120-Nrf1) and their regulation by Nrf2, may pave the way for development of novel therapies against CRPC cells. In addition, determination of the expression and localization of p65-Nrf1, p120-Nrf1, and Nrf2 may also be a useful biomarker for identifying patients with aggressive cancer that may become resistant to ADT.

## Supporting Information

Figure S1 **Effect of p65-Nrf1 overexpression on PSA and TMPRSS2 gene expression.** LNCaP cells were transfected with either control vector (pcDNA3.1) or the p65-Nrf1 expression vector (p65-Nrf1-V5-His). Cells were stimulated with DHT (10 nM) and total RNA were isolated at 24 hr to measure the expression of two AR-regulated genes, TMPRSS2 and PSA, by qRT-PCR. Data (Ct values) were normalized to GAPDH mRNA levels is respective samples and fold changes in TMPRSS2 and PSA mRNA levels are shown. (n = 2; *, p<0.05; **, p<0.01).
(TIF)

Figure S2 **Effects of Nrf1 or Nrf2 on AR gene expression and AR nuclear localization.** Modulatory effects of p65-Nrf1 and Nrf2 on AR gene expression and nuclear AR levels were measured in LNCaP and C4-2B cells. The AR mRNA levels were determined by qRT-PCR and nuclear AR protein was measured by western immunoblotting. Fold changes in relative AR gene expression after, (A) p65-Nrf1 overexpression in LNCaP cells, (B) Nrf1 knockdown by siRNA in C4-2B cells, or following Nrf2 overexpression in either LNCaP (E) or C4-2B cells (F) are shown. For qRT-PCR studies, all Ct values were normalized to their corresponding GAPDH levels (n = 2). Immunoblotting of nuclear AR was carried out after (C) p65-Nrf1 overexpression (pCMV-Nrf2) in LNCaP cells or (D) following Nrf1 knockdown (siRNA) in C4-2B cells. AR nuclear levels were normalized to TBP levels in each sample (n = 2).
(TIF)
