# Supplementary figures and images for: Nrf1 and Nrf2 Transcription Factors Regulate Androgen Receptor Transactivation in Prostate Cancer Cells
Source: PLoS One. 2014 Jan 22;9(1):e87204. doi: 10.1371/journal.pone.0087204 (PMC3899380; doi:10.1371/journal.pone.0087204)

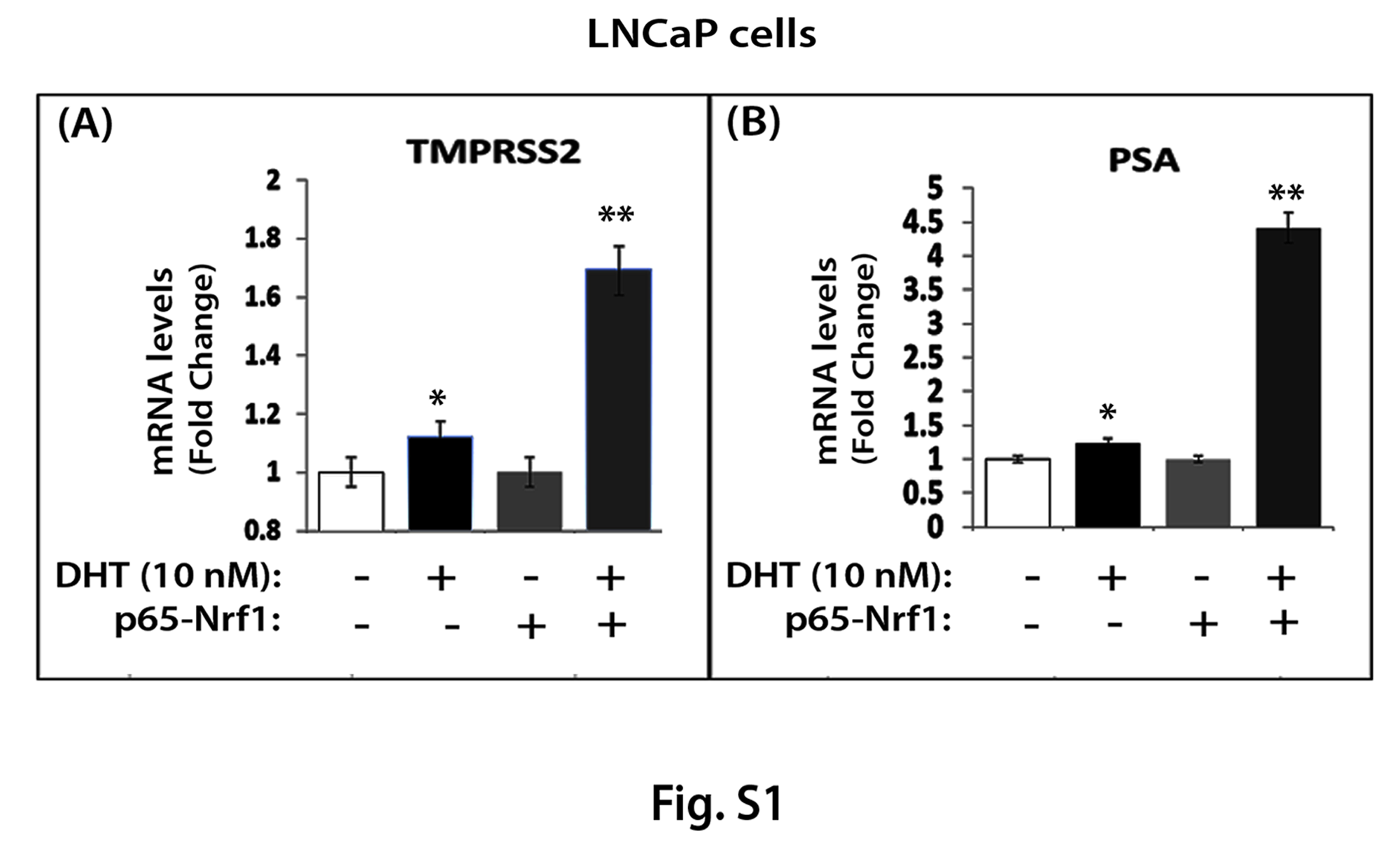

Supplement: Figure S1 — Effect of p65-Nrf1 overexpression on PSA and TMPRSS2 gene expression. LNCaP cells were transfected with either control vector (pcDNA3.1) or the p65-Nrf1 expression vector (p65-Nrf1-V5-His). Cells were stimulated with DHT (10 nM) and total RNA were isolated at 24 hr to measure the expression of two AR-regulated genes, TMPRSS2 and PSA, by qRT-PCR. Data (Ct values) were normalized to GAPDH mRNA levels is respective samples and fold changes in TMPRSS2 and PSA mRNA levels are shown. (n = 2; *, p<0.05; **, p<0.01). (TIF) [file pone.0087204.s001.tif]

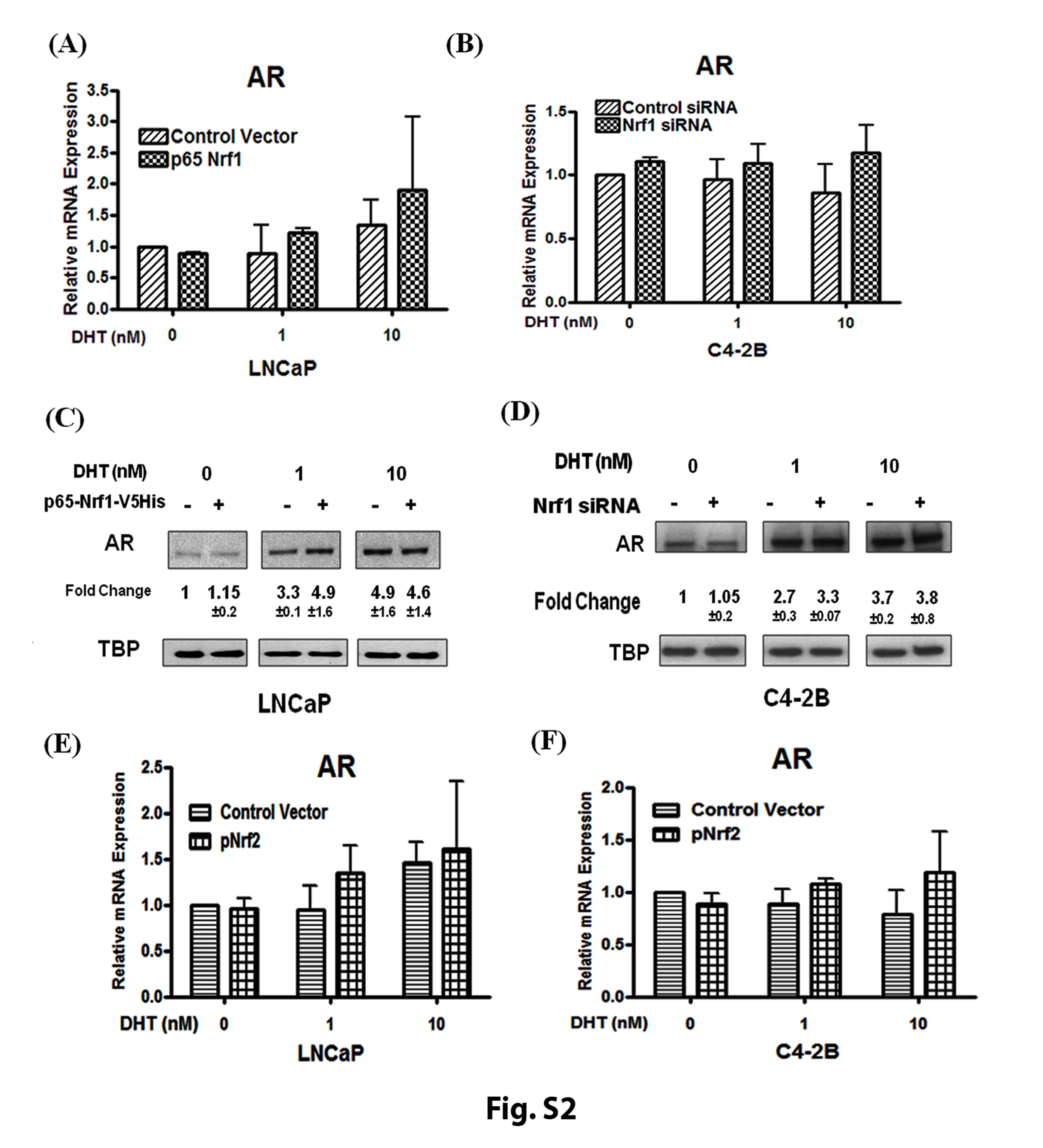

Supplement: Figure S2 — Effects of Nrf1 or Nrf2 on AR gene expression and AR nuclear localization. Modulatory effects of p65-Nrf1 and Nrf2 on AR gene expression and nuclear AR levels were measured in LNCaP and C4-2B cells. The AR mRNA levels were determined by qRT-PCR and nuclear AR protein was measured by western immunoblotting. Fold changes in relative AR gene expression after, (A) p65-Nrf1 overexpression in LNCaP cells, (B) Nrf1 knockdown by siRNA in C4-2B cells, or following Nrf2 overexpression in either LNCaP (E) or C4-2B cells (F) are shown. For qRT-PCR studies, all Ct values were normalized to their corresponding GAPDH levels (n = 2). Immunoblotting of nuclear AR was carried out after (C) p65-Nrf1 overexpression (pCMV-Nrf2) in LNCaP cells or (D) following Nrf1 knockdown (siRNA) in C4-2B cells. AR nuclear levels were normalized to TBP levels in each sample (n = 2). (TIF) [file pone.0087204.s002.tif]
